# The Challenges of Transition From Donor-Funded Programs: Results From a Theory-Driven Multi-Country Comparative Case Study of Programs in Eastern Europe and Central Asia Supported by the Global Fund

**DOI:** 10.9745/GHSP-D-18-00425

**Published:** 2019-06-24

**Authors:** George Gotsadze, Ivdity Chikovani, Lela Sulaberidze, Tamar Gotsadze, Ketevan Goguadze, Nertila Tavanxhi

**Affiliations:** a Curatio International Foundation, Tbilisi, Georgia.; b UNAIDS, Geneva, Switzerland.

## Abstract

**Introduction:**

In the era of declining development assistance for health, transitioning externally funded programs to governments becomes a priority for donors. However, the process requires a careful approach not only to preserve the public health gains that have already been achieved but also to expand on them. In the Eastern Europe and Central Asia region, countries are expected to graduate from support from the Global Fund to Fight AIDS, Tuberculosis and Malaria in or before 2025. We aim to describe transition risks and identify possible means to address them.

**Methods:**

Using a theory-based conceptual framework—Transition Preparedness Assessment of Tuberculosis and HIV/AIDS programs—we investigated transition-related challenges through a health systems lens in 10 countries of the Eastern Europe and Central Asia region during 2015–2017. Study findings were derived from systematic collection of quantitative data on socioeconomic indicators and disease epidemics as well as qualitative data from in-depth interviews with 264 stakeholders. These findings were then compared with other donor transition experiences documented elsewhere.

**Results:**

We found numerous common transition challenges, such as poor monitoring of a country's macroeconomic performance along with weakness in estimating financial needs for successful transition; limited political will of governments to replace donor-funded programs; punitive legislation criminalizing certain behaviors and constraining the government's ability to allocate funds and contract civil society organizations essential to providing services for key populations; limited coordination function of governments and weak decision-making power of coordinating mechanisms obscuring the latter's future role; and inadequate function of national procurement and supply chain management systems undermining an uninterrupted supply of quality-assured drugs and commodities. These challenges are compounded by the risks related to health workforce management leading to specialist shortages and/or inadequately skilled and qualified professionals and by limited funding for critical surveillance activities.

**Conclusion:**

The complex and multidimensional transition process requires a multipronged approach through well-planned collective and coordinated responses from global, bilateral, and national partners in coming years. Other similar transition processes may provide guidance. Although no “one-size-fits-all” approach exists, previous experiences highlight a need for both early planning and monitoring of the transition along several key dimensions. Issues that could threaten the maintenance of health gains include ongoing stigma against key populations; continued heavy reliance on external funding in some countries, especially for preventive services; the institutional viability of the country coordinating mechanisms; and emerging difficulties with procurement of quality drugs at reasonable prices.

## Abstract (precis)

Transitioning from donor funding toward domestic financing for HIV and TB programs in Eastern Europe and Central Asia presents major challenges. It will require a substantial multipronged approach through well-planned collective and coordinated responses from global, bilateral, and national partners.

## INTRODUCTION

During past decades, development assistance for health (DAH) increased substantially and fostered progress toward global health goals.^1^ Increased investments in health have helped countries to improve maternal, newborn, and child health, and to reduce the spread of HIV/AIDS, malaria, tuberculosis (TB), and other major infectious diseases.^2^^,^^3^ These investments were channeled through bilateral and multilateral agencies, as well as through global health initiatives such as Gavi, The Vaccine Alliance; the Global Fund to Fight AIDS, Tuberculosis and Malaria (Global Fund); and so forth.

In the field of HIV/AIDS, TB, and malaria, the Global Fund, among others, was pivotal in achieving public health gains by investing close to US$38.7 billion or approximately 9% of DAH during 2002–2016.^4^ These investments helped to enhance the national coordinating structures in charge of national responses to TB, HIV/AIDS, and malaria; advance public-sector capacity; mobilize civil society and community organizations and engage them in service delivery especially for the most at-risk and vulnerable populations; and expand and scale up preventive, diagnostic, curative, and supportive interventions. Above all, these investments raised public awareness about epidemics and promoted approaches based on human rights.

Following the 2008 global financial crisis, DAH levels stagnated, and declining trends have been observed since 2013.^5^ This decline has triggered debates within the donor community about the gradual transition of donor-funded programs to country ownership.^6^^–^^10^ These discussions have also affected the Global Fund, which led the Executive Director to raise the following concern with the Board^11^:

*With some humility, we can admit that in development work, including global health, there have been a lot of exits but not many successful transitions. Programmatic and financial sustainability takes time, planning and a balanced portfolio of trades and investments along the development continuum.*

In other words, the public health gains achieved by recipient countries seem to be at risk unless the transition from donor support in general, and from the Global Fund in particular, is well planned and executed. This issue takes on even greater importance in the context of the Sustainable Development Goals, which include commitments to universal health coverage^12^ and renewed commitments to Alma Ata.^13^^,^^14^

Consequently, we decided to examine the potential transition challenges in countries in Eastern Europe and Central Asia (EECA) that are expected to graduate from Global Fund support in or before 2025.^15^ Using theory-based comparative case studies from 10 countries (Armenia, Belarus, Bulgaria, Georgia, Kosovo, Kyrgyzstan, Moldova, Turkmenistan, Ukraine, and Uzbekistan), we investigated the programmatic areas within a broader country context that could be at greatest risk during transition. We hope these findings will facilitate discussions on potential solutions going forward as well as inform discussions around transition within the Global Fund, among donors and recipient countries.

Using case studies from 10 countries, we investigated the programmatic areas that could be at greatest risk during transition from Global Fund support.

### The Challenges of EECA

Since 2000, EECA has made significant progress addressing the challenges posed by a growing epidemic of TB and HIV/AIDS. However, the threats remain and the region requires even greater attention as countries head toward transitioning from donor support. In this section, we briefly describe the most significant epidemiological and other trends that need attention, highlighting the importance of the region from an epidemiological perspective.

Although the rate of new HIV infections is decreasing globally, it more than doubled in EECA between 2006 and 2015. Due to the low level of testing coverage, almost a third of the people infected are not aware of their HIV status.^16^ The HIV epidemic is concentrated predominantly among key populations (KPs) that are driving the growth of the epidemic—primarily, people who inject drugs followed by men having sex with men (MSM). While support from donors, especially the Global Fund, has led to significant progress in developing, delivering, and scaling up preventive, diagnostic, curative, and support services for KPs, the coverage rates of HIV prevention programs within the region are still low.^17^ According to United Nations Office on Drugs and Crime,^18^ almost a quarter of the people injecting drugs around the world reside in the EECA region, or approximately 2.9 million people. However, coverage with opioid substitution therapy remains below 5% in all but 3 states, and access to needle and syringe programs, while variable across countries, remains below the recommended 200 clean needles and syringes per person who injects drugs per year.^16^

While support from donors has led to significant progress in services for key populations, the coverage rates of HIV prevention programs are still low.

Despite the efforts of the past decade to scale up treatment coverage, only 21% of people living with HIV/AIDS (PLHIV) in EECA were receiving treatment in 2015, which is far below the global average of 53%. Thus, the rate of new infections continues to outpace antiretroviral therapy enrollment^19^ and undermine the goal to end AIDS as a public health threat by 2030.

HIV prevalence data for MSM are variable and grossly misleading for the region due to weak surveillance systems.^20^ The reported prevalence among MSM in some countries is as high as 20.7% in Georgia and as low as 0.8% in Armenia.^21^ High HIV rates are compounded by variable rates of self-reported condom use, ranging from 49% in Moldova to 81.6% in Kyrgyzstan.^22^ The percentage of MSM reporting using a condom the last time they had anal sex with a male partner varies from 80.4% in Armenia to 61.2% in Moldova,^21^ with low usage facilitating the spread of infection.

Fighting societal stigma and implementing HIV response are impeded by conservative legislation and political and cultural barriers related to same-sex relationships, drug use, and sex work. These challenges often drive both behavior and services underground, reducing the scale and impact of disease programs. Stigma and discrimination also continue to hinder access to HIV prevention, treatment, and care services for KPs, thereby exacerbating social inequalities.^16^^,^^17^

The situation with TB is similarly poor. Even though the broader European region (including EECA) has had declining TB rates since 2000, which has led to a reduced TB burden, multidrug-resistant TB (MDR-TB) has emerged as a substantial public health threat. Nine of 30 countries with the highest MDR-TB burden in the world are in EECA, representing about 20% of the global MDR-TB burden (approximately 350,000 individuals). The proportion of MDR-TB cases among new and previously treated TB cases in the region is significantly above the global average, with 19% in new and 55% in previously treated cases, compared with 4.1% and 19%, respectively, as of 2016.^23^ Despite universal treatment coverage for TB and MDR-TB, the treatment success rate in the region is below regional and global targets, which indicates a need to improve treatment program performance.

The European region has had declining TB rates since 2000, but multidrug-resistant TB has emerged as a substantial public health threat.

High rates of TB and HIV co-infection plague the region. Furthermore, TB was the most common AIDS-defining illness in the EECA region in 2015, and the number of incident TB cases co-infected with HIV almost doubled (from 5.5% to 9%) between 2011 and 2015.^24^ TB remains a significant cause of death among PLHIV: the rate of TB-related deaths among PLHIV increased by 3.6% annually between 2011 and 2015.^25^ Action is required. Civil society as well as communities in EECA are appealing to the West to pay adequate attention to these mounting threats as donors consider transitioning the region away from external support.^26^^–^^28^

Managing donor transitions responsibly is crucial to ensure that the public health gains attained with donor support are not only preserved but also expanded, which is essential to adequately deal with the TB and HIV threats in the region.

## METHODS

Following Bennett et al.,^29^ we define transition as the formal handing over of a donor-funded health program to one or more local partners in a way that ensures critical elements of the program are sustained over time. Given how central the notion of transition is to sustainability and the long-term effects of the entire development enterprise, surprisingly little literature and relatively limited empirical evidence exist with regard to what constitutes good transition practice^29^ and what needs to be done during the process. Transition is increasingly seen as a process rather than an outcome^30^ because the programs evolve through complex adaptive systems, while responding to changing contexts^31^; as a result, the activities implemented during transition need to meet ever-changing needs. To achieve sustainability after donor funding has ended and to retain or expand the public health gains that were achieved during donor support, the transition process must be adequately understood and managed. Although much of the research in the field of transition is retrospective, we decided to undertake an evaluation of the transition issues inside a group of 10 countries *before* transition based on our case studies arising from short-term consultancy work.^6^^,^^32^ We examined TB and HIV/AIDS programs supported by the Global Fund, with the exception of the HIV/AIDS program in Turkmenistan, and our evaluation of 19 programs in 10 countries has informed the findings of the current study.

Curatio International Foundation, with the financial support from the Global Fund, developed a theory-based conceptual framework for a transition preparedness assessment (TPA) of Global Fund-supported programs (Figure). The TPA framework builds on existing sustainability frameworks,^31^^,^^34^^–^^36^ including the U.S. President's Emergency Plan for AIDS Relief (PEPFAR) Sustainability Index and Dashboard,^37^ using a health systems lens. The TPA framework looks at external and internal environments, their subdomains, and components such as economic and political support, financial and human resources, information systems, governance, accountability, service delivery, organizational capacity and state of transition planning (the TPA framework components are described in the Figure). Using quantitative and qualitative information/indicators, the TPA framework helps to identify the sustainability-related issues that require attention during transition. The framework was operationalized as a tool that applies a scoring system for each indicator under a component. Each component is assigned a risk category for transition (high, moderate, or low) based on the measurement of its indicators. A more detailed description of the TPA tool methodology is available elsewhere.^38^ The data for this article were collected during the period 2015–2017 from 10 countries in the EECA region using the TPA framework and tool. Although the TPA tool has also been used in Jamaica, Morocco, and the Philippines, we maintain a regional focus for this paper on EECA, specifically Armenia, Belarus, Bulgaria, Georgia, Kosovo, Kyrgyzstan, Moldova, Turkmenistan, Ukraine, and Uzbekistan.

**FIGURE:**
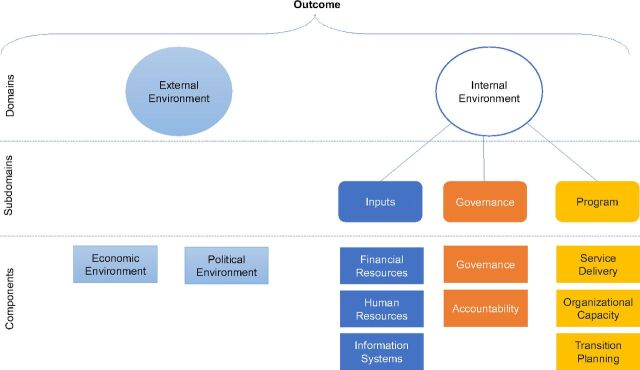
Transition Preparedness Assessment Framework and Components Source: Amaya AB et al. (2015).^33^

Using quantitative and qualitative information, the TPA framework helps to identify the sustainability-related issues that require attention during transition.

To apply the TPA tool, we used a mixed-methods approach consisting of desk review, quantitative data collection from public databases, and qualitative data collection through in-depth interviews. The desk review focused on legislative and policy documents, program performance, evaluation/reviews, program expenditure reports, and other types of reports. Quantitative data on socioeconomic indicators and disease epidemics were collected from databases belonging to the World Bank, the Joint United Nations Programme on HIV and AIDS (UNAIDS), and the World Health Organization (WHO). In-depth interviews (using standard guidance/tools adjusted to specific country contexts) were conducted with key stakeholders who were selected through snowball sampling including government officials; the principal recipients of Global Fund grants; national HIV and TB programs representatives; members of civil society organizations (CSOs), international NGOs, and UN agencies; and representatives of the Global Fund secretariat in Geneva. A total of 264 respondents were interviewed (see Supplement Table 1 indicating the number of country representatives). To ensure robustness of the data, the country findings were triangulated across the different data sources, where possible. Finally, the TPA framework helped to systematically extract and compare data/information across the country case studies to identify the common program areas exposed to transition risks.

By revealing the more generalizable trends across countries, we intend to (1) describe the areas at greatest risk during the transition, and (2) contribute to global knowledge about the most expected transition challenges in order to enrich the debates around transition planning by donors/funders and countries.

We intend to describe the areas at greatest risk during the transition, and contribute to global knowledge about transition challenges.

## RESULTS

In this section, we organize the findings around the external environment, and then present the results concerning the internal/program environment with its subdomains and components. The Table provides a summary matrix of the most common barriers of transition found across the studied countries in the EECA region. The barriers are structured by TPA components and categorized by high, moderate, or low risk for transition. More granular information by country is provided in Supplement Table 2.

**TABLE.**
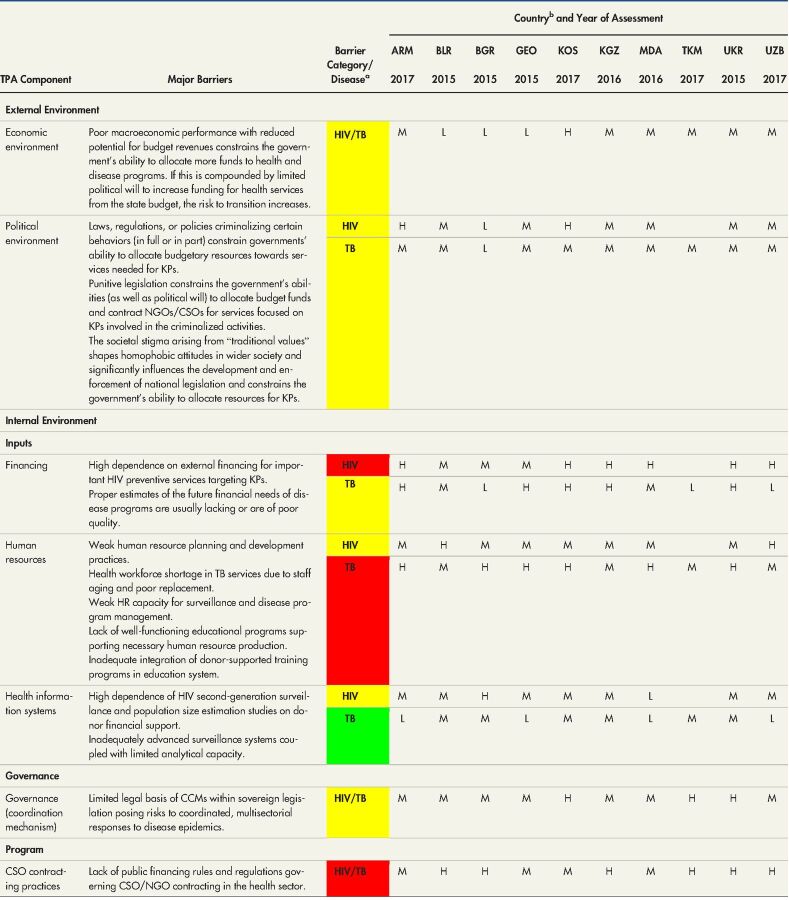

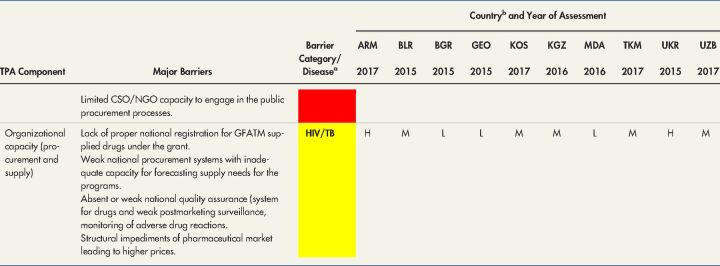
Summary Matrix of Transition Barriers Across the Study Countries

Abbreviations: ARM, Armenia; BLR, Belarus; BGR, Bulgaria; CCM, country coordinating mechanisms; CSO, civil society organization; GEO, Georgia; GFATM, Global Fund to Fight AIDS, Tuberculosis, and Malaria; HR, human resources; KGZ, Kyrgyzstan; KOS, Kosovo; KP, key population; MDA, Moldova; TB, tuberculosis; TKM, Turkmenistan; TPA, transition preparedness assessment; UKR, Ukraine; UZB, Uzbekistan.

a The red color denotes high risk for transition or existence of many or significant barriers, yellow denotes moderate risk for transition or existence of few barriers, and green indicates a low risk for transition or an absence of major barriers.

b For country-level findings, the risk categories are indicated by letters: H for high risk, M for moderate risk, and L for low risk for transition. A blank cell indicates that the topic was not studied.

### Economic and Political Environment

The economy determines a country's capacity for national budgets, including funding for the health sector and disease programs. The economies of the countries studied performed well during 2010–2016, although the average annual per capita GDP growth rate varied from 0.47% (Ukraine) to 7.8% (Turkmenistan).^39^ During the same period, government revenues (not including grants) averaged 28.9% of GDP across the sample with significant variability: the lowest proportion (23.1% of GDP) occurred in Armenia, while the highest (35% of GDP) was in Ukraine.^40^ Besides the variable macroeconomic performance, the political will of governments to fund health in general and disease-specific programs in particular is a prerequisite to ensure that they take responsibility for replacing donor funding during the transition. The importance of a nation's health in public financing is highly variable when considered as the proportion of the state budget devoted to health. This proportion ranged between 5% in Georgia and 13.5% in Belarus in our sample (based on averages for the 2010–2014 period). At the same time, the share of public spending in total health expenditure ranged from 18.7% in Georgia to 71.5% in Belarus (average figures for 2010–2014).^41^ The study also found that the estimations of future financial needs for the national programs were either of poor quality and/or not available.

Laws and regulations criminalizing specific behaviors are another important component of the external environment because they could pose significant challenges to the transition process. All countries in this study, except Bulgaria, criminalize the behaviors associated with KPs to varying degrees. In some countries such as Armenia, Belarus, and Moldova, illicit drug consumption is a criminal offense. In Belarus, changes in the legislation have created additional barriers and reinforced stigma and discrimination, such as mandatory registration of all drug users in an electronic database accessed by law enforcement body, obligatory disclosure of HIV status when a person seeks medical care, and compulsory HIV testing. In Uzbekistan, homosexual sex is still criminalized and sex work is subject to an administrative fine.

### Financing of Disease Programs

The dependence of the programs on donors is sizable but also variable across countries. For example, the HIV/AIDS program in Moldova was 68% externally funded for 2015, while in Uzbekistan, the HIV/AIDS program received only 29% from donors during the same year. We found that the TB program had less dependence on external funding, ranging from 15% in Turkmenistan and in Uzbekistan (2015) to 51% in Kyrgyzstan (2016).^42^ Dependency on external support becomes even more apparent when the disease program components are considered. For example, KPs are prioritized in almost all national HIV/AIDS strategic plans of the sample countries, but harm reduction and other prevention activities largely rely on external (Global Fund) financial support. Furthermore, public spending on KPs over past years did not grow, and if any increases occurred, they were marginal.

In all cases except Moldova, low threshold services are not funded by public sources; Belarus, Moldova, and Ukraine provide little cofinancing from the national budget for opioid substitution therapy services, and only Georgia and Bulgaria fund these services predominantly from the national budget. Moreover, all other countries entirely depend on the Global Fund for funding opioid substitution therapy service provision. In sum, all countries exhibit significant dependence of preventive interventions from external sources. Similarly, TB programs in 10 countries depended heavily on Global Fund support for drugs especially for second-line drugs and diagnostics (Supplement Table 2). By 2016, close to 40% of TB allocations from the Global Fund went for pharmaceutical products, another 10% for nonpharmaceutical products, and 5% for equipment. However, TB programs planned to reduce external funding by increasing national budget spending starting from 2017,^43^ but the actual results still have to be validated.

All countries exhibit significant dependence of preventive interventions from external sources.

### Social Contracting

Continuing engagement of NGOs/CSOs emerged as one of the critical impediments in the transition process in all countries. During the past decade, Global Fund and donor funding to NGOs/CSOs helped to increase their role in delivering critical preventive, care, and support services, primarily to KPs. Their role increased more prominently in the HIV programs than in TB, for which active CSO engagement is relatively recent but growing. Most NGO/CSO-delivered services are externally funded and face the threat of reductions in Global Fund support. Such reductions would decrease the volumes of delivered services by NGOs/CSOs unless state budgets pick up the tab and/or greater efficiency in service delivery is achieved.

According to Aceso Global, social contracting is the process by which government resources are used to fund entities that are not part of government (e.g., CSOs) to provide services. Social contracting may have various names and slightly different mechanisms between countries. Regardless of the terminology used, social contracting mechanisms typically involve a legally binding contract, in which the government agrees to pay a CSO for services rendered and the CSO agrees to provide certain deliverables in exchange.^43^

Deeper examination of the context for social contracting revealed that all the countries in our sample have enacted legislation that allows government contracting of NGOs/CSOs. Furthermore, all the countries in our sample practice civil society contracting in sectors other than health. However, within our sample, only Armenia, Georgia, Kosovo, and Moldova use social contracting in the health sector. Belarus introduced changes to the laws allowing for social contracting of NGOs for prevention of HIV/AIDS and other communicable diseases only in mid-2017, while other countries have not yet tried NGO contracting. Thus, while NGO/CSO contracting frameworks are included in the national legislation, in most instances the mechanisms either are not relevant for procuring preventive and other services in the health sector and/or they do not specify the necessary details for the allocation and/or disbursement of public funds. Examples include the lack of specific national standards or guidelines defining services currently being delivered by NGOs/CSOs; the lack of methodology (and capacity) necessary for estimating the budget requirements for the services and/or for evaluating the adequacy of quoted prices in the bids; the lack of clear tendering procedures, bid selection criteria, and contracting terms and conditions (including programmatic and financial reporting requirements for NGOs/CSOs); and the lack of guidelines/procedures for monitoring the volume and quality of delivered services. In addition, few CSO/NGOs in the countries have sufficient financial and technical capacity to engage in public procurement processes.

All countries studied have legislation allowing government contracting of NGOs/CSOs, but few use such contracting in the health sector.

### Governance and Coordination Function

Without strong governance and cross-sectoral coordination arrangements in place, managing the complex process of transition will be difficult. A lack of vision for continuing intersectoral coordination that ensures civil society engagement during and after transition emerged as an area of concern in our review. While almost all stakeholders highlighted the importance of country coordinating mechanisms (CCMs), the feasibility of maintaining CCMs in the post-Global Fund era is questionable due to the currently weak institutional placement of CCMs in sovereign governance structures. For example, a CCM is placed under the central government only in Belarus, Bulgaria, and Uzbekistan. In other countries, CCMs are created by government resolutions but are largely placed under the ministries of health, and other sectors typically do not attend the meetings. Furthermore, most respondents questioned the ability of CCMs to make decisions and ensure their implementation because CCM decision-making powers are not aligned with the sovereign governance systems/rules. These concerns were further exacerbated by the CCMs' almost complete dependence on Global Fund funding, without a clear vision for how they would be supported financially in the post-Global Fund era.

The feasibility of maintaining country coordinating mechanisms post-transition is questionable due to their weak institutional placement in sovereign governance structures.

### Human Resources

Weak human resource planning and development practices along with a shortage of TB specialists due to staff aging and poor replacement are challenges that pose risks to transition if not addressed adequately. Almost all the studied countries are experiencing active labor migration; have failed to plan for an adequate number of health professionals; lack deployment and staff motivation policies; and have health workforce shortages affecting provision of TB care. In all the countries, at least 20% of TB specialists are in the preretirement and retirement age groups. In Georgia, the proportion is 30%, while it is even higher in Kyrgyzstan (44%). Due to low financial motivation and professional risk exposure, younger people throughout the region are not willing to pursue this profession. Additionally, HIV and TB training courses that were developed with the support of development partners are not yet fully integrated into national undergraduate, postgraduate, or continuing professional development programs. The exception is Moldova, where all levels of medical education have been updated according to the latest guidelines. Some countries have sporadically updated continuing medical education curricula, while undergraduate and residency programs lag behind. The countries lack a repository of all necessary training materials and qualified master trainers. If these issues are not addressed during transition, once external funding ends, the medical education system will produce health professionals without adequate professional knowledge and skills.

### Procurement and Supply Chain Management

Considering transition-related procurement and supply chain management issues under the “organizational capacity” component of the TPA framework (Figure), we found that an uninterrupted supply of quality-assured drugs and diagnostics seems to be at risk during and after the transition. Countries in the sample still rely on Global Fund procurement mechanisms such as voluntary pooled procurement—a Global Fund strategic initiative that aggregates order volumes on behalf of participating grant recipients to negotiate prices and delivery conditions with manufacturers, mainly for second-line antiretroviral (ARV) drugs. For TB drugs and commodities, they also rely on the global drug facility (GDF), a procurement mechanism that is the largest global provider of quality-assured TB medicines, diagnostics, and laboratory supplies to the public sector. Some countries, such as Belarus, Kyrgyzstan, and Armenia, have already established alternative supply channels using public financing, albeit with emerging challenges. Specifically, purchasing drugs and commodities through alternative and/or national suppliers, have resulted in the following:

- **Prices higher by 80% or more for ARV and TB drugs and diagnostics.** Belarus paid higher prices for ARV drugs; depending on the medicine, the prices were 2 to 8 times those of the Global Fund. In Uzbekistan, regional HIV centers purchased test systems through a decentralized, local procurement process at prices that were 70%–80% higher than those of the Global Fund. Ukraine paid a higher price for ARV drugs prior to amending legislation in 2015. These developments are not unique to HIV and TB or to the EECA region; they were also observed in other countries that had graduated from the Global Fund or Gavi, when price increases were significant for purchases through local/regional suppliers.^8^^,^^44^ Price increases naturally demand higher amounts of public funds and thus emerge as a threat to transition and sustainability.
- **Questionable quality of the commodities supplied, especially drugs, which could negatively affect treatment outcomes and lead to drug resistance.** When dependent on Global Fund grants, and therefore on the commodities supplied through quality-assured international systems, countries almost always used one-time import waivers to procure drugs as opposed to obtaining proper market authorization following national legislation. Consequently, quality-assured drugs supplied through Global Fund systems were not registered in the graduating markets, and when countries moved to public financing and applied sovereign procurement rules, they were frequently left with only local suppliers that may not have had quality-assured products. This challenge is compounded by weak or absent national quality assurance (QA) systems for drugs, coupled with weak postmarketing surveillance and monitoring of adverse drug reactions. While all EECA countries have QA requirements for pharmaceuticals, usually based on international recommendations, local stakeholders question the degree of successful implementation and the efficiency of these systems.
- **An interrupted supply of drugs.** Numerous factors adversely affected drug supply, including poor planning and quantification of the needed commodities (most often due to a lack of capacity); delays in delivery from local suppliers winning national tenders; administrative-bureaucratic challenges arising from national public procurement and financing rules; and little to no interest from pharmaceutical companies to participate in public tenders due to the countries' small market size or other reasons, such as corrupt practices.

An uninterrupted supply of quality-assured drugs and diagnostics seems to be at risk during and after the transition.

Thus, national procurement of drugs and commodities require specialized market knowledge, institutions, and skills that still need to be built or enhanced in some graduating countries if the quality of supplied drugs and commodities is to be secured during and after the transition.

### Health Information Systems

Health information systems that produce critical epidemiological and program data are pivotal for adequate national response planning and management. External assistance throughout the past decade has been critical in building and developing these systems for evidence-based decision making. Most countries have moved towards electronic TB information systems, supported by external assistance, but HIV information systems lag behind. However, the ability of these systems to capture the comprehensive data necessary for evidence-based planning, nationwide coverage, and application in practice varies across countries.

The ability of health information systems to capture the comprehensive data necessary for planning, nationwide coverage, and practice varies across countries.

Repeated waves of bio-behavioral surveillance (BBS) studies among KPs, and more recently population size estimation (PSE) studies have been critical for measuring national HIV/AIDS program outcomes. They also offer important information for advocacy and program management. Although the methodologies have variable rigor, these studies are vital for tracking disease prevalence and population behavior, especially among KPs. Countries in totality depend on external financial support to implement BBS and PSE studies.

To conclude, the findings reveal that Global Fund program transitions are expected to face multiple risks, and therefore a more carefully planned approach to transition is required.

## DISCUSSION

The evaluation of transition processes in our sampled countries revealed that transition processes are complex and multidimensional, encompassing numerous domains as well as actors at all levels within and outside the health system. In this section, we discuss the differences and similarities of major transition challenges and relate the lessons learned from the countries of the EECA region and countries that have already transitioned from Global Fund support. We also discuss countries' experiences of graduating from the support of other bilateral and multilateral donors, including the United States Agency for International Development (USAID), the Bill & Melinda Gates Foundation, and Gavi. Such comparisons help to identify potential solutions that have worked in other parts of the world and could be instrumental for the Global Fund going forward.

Transition processes are complex and multidimensional, encompassing numerous domains as well as actors within and outside the health system.

The available literature suggests that a collaborative and coordinated process between donors and countries is important in planning for transition.^10^^,^^32^^,^^44^^–^^47^ This approach helps to generate political commitment from the government and stronger buy-in from national stakeholders who help plan and manage successful transition by alleviating or mitigating potential negative consequences. The process includes 4 essential elements^48^ which are described in greater detail later in this section:

- Early planning with the government to reach a mutually acceptable and time-bound transition plan divided into phases with clear milestones, which allows for sufficient time for any adjustments and corrections necessary to proactively mitigate existing or emerging risks
- Aligning donor-funded program components with government structures and funding modalities before transition
- Building government capacity through active technical assistance and management support, and budgeting for adequate support during and after the transition
- Developing and using a framework for monitoring the transition process along with a mechanism for ensuring mutual accountability between a donor and a country

A transition plan represents a necessary instrument for securing a government's political commitment to use its funds and capabilities and gradually replace donor-funded services, commodities, and management responsibilities during and after graduation. Our findings corroborate findings in the literature that indicate achieving successful transition requires close monitoring of a country's macroeconomic performance and its fiscal space, along with the current and expected health sector and disease spending needs. We also found that accurate and reliable estimates for the future financial needs of disease programs are usually lacking or are of poor quality, which will likely negatively affect adequate financial planning for transition. Therefore, estimating national financial needs during and after donor graduation, while simultaneously monitoring a country's fiscal space, seems necessary when making transition decisions and/or planning for the duration of the transition process. The literature suggests that transition planning requires forecasts of expected program cost at least 5–10 years into the future in order to account for realistic and not purely aspirational programmatic goals.^48^

A transition plan supports a government's commitment to use its resources to replace donor-funded services, commodities, and management responsibilities.

However, political commitment should not only be financial; it also has to include an obligation to enact legislative and regulatory changes to address the barriers. Apparently, most diagnostic and treatment services (except second-line drugs for TB patients) are already funded and delivered by the governments. However, preventive services are highly dependent on external support.

Political commitment should not only be financial; it also has to include making legislative and regulatory changes to address the barriers.

In addition, we found that punitive legislation constrains governments' ability and political will to allocate budget funds and contract NGOs/CSOs for services focused on KPs. It is important to recognize that “traditional values” influence the development and enforcement of punitive legislation that makes KPs more marginalized and vulnerable to HIV. Unless such legislation and regulations are amended during the transition process, achieving sustainable handover of certain preventive services would be very difficult. Also, punitive legislation imposes access barriers to services, unless services are delivered by CSOs in a friendly environment for KPs. Examples include delivering needle exchange services where illicit drug use is criminalized; offering services to sex workers and MSM, including condom distribution, where criminal or administrative liability exists for these individuals; and requiring mandatory parental consent for adolescents for HIV testing.

Most attempts to build political commitment must be targeted at national governments because the budgets, laws, policies, and regulations that can sustain a health program in the long term often flow from governments and are closely interlinked.^49^ We found that CCMs in the studied countries lack adequate placement in the government hierarchy, which leads to weak coordination and decision-making power. In addition, their future role after transition is vague. The experiences of countries that have transitioned from Global Fund support suggest that CCM and their coordination function fades after transition and limits CSO involvement in the decision-making process.^50^ We speculate that if CCM funding was shifted to the Ministry of Health budget, it would still be uncertain whether the secretariats could maintain their independence and cross-sectoral coordination roles. Consequently, the Global Fund practice of seeking CCM-approved transition plans, in a context in which the legal powers of current CCMs and their abilities after transition are uncertain, may require rethinking.

Transition plans found to be central to sustainability have included the following elements: strategic prioritization of critical program areas; budgeted recommendations for action; and a clear timeline and phases for graduation, with associated benchmarks and indicators to assess progress.^10^^,^^51^ Planning for transition proved useful for the Avahan program in India,^7^ Gavi graduation,^8^ and USAID-supported family planning programs.^6^^,^^10^ However, developing a transition plan is both a technical and a political process that requires adequate time for development and proportional investments. Such a plan requires transition risk analysis and thorough planning; fiscal space analysis and adequate estimation of the required resources through different budget scenarios^10^^,^^44^; agreement for equitable sharing of the financial burden between donors and the country in question during the transition; and planning for the integration of disease programs with national funding streams. Most importantly, it also requires a process of intensive dialogue and the establishment of transparent accountability mechanisms between domestic and international funding organizations.^32^^,^^48^

Before graduation from donor support, a progressive reduction of dependency on external support is needed along with the alignment of donor-funded program components with government structures and funding modalities. Based on experiences from other programs, these steps may require coordinated program harmonization with existing services during the transition. This harmonization can include adapting program services and implementing arrangements for planning, management, financial reporting, and monitoring and evaluation, to foster integration with the national program or host environment.^52^ To facilitate program transition, technical, managerial, and cost elements of programs need to be aligned with government norms.^32^ As noted in the 10 countries studied, Global Fund-supported programs operated their own supply line of quality-assured drugs and diagnostics, which were imported to the country with one-off waivers instead of following national regulations and securing proper market authorization. Critical surveillance activities such as BBS and PSE studies were solely donor funded instead of being integrated as a routine component of national program surveillance supported by the government; and CSO/NGOs to deliver critical services to KPs were funded from external sources. Challenges in so-called “social contracting” were prominent in our sample and are also well described elsewhere in the literature.^45^^,^^53^^–^^55^ Funding NGOs/CSOs from a state budget is challenging on several counts: (1) legal and societal barriers limit governments (and their political will) in allocating and spending budget funds on services, primarily focused on KPs; (2) public financing mechanisms/regulations allowing governments to contract NGOs/CSOs using budget funds in the health sector are lacking; and (3) NGOs/CSOs have a limited capacity to engage in government procurement processes to manage public funds due to national regulations. Thus, alignments during transition are multipronged and require durable mechanisms (legislative, procurement systems, or otherwise) to be established for the continuous supply of quality-assured commodities during and after transition with national funding. The process must include establishing CSO/NGO contracting systems/mechanisms/regulations to ensure uninterrupted service delivery to KPs after Global Fund graduation. Further, surveillance activities currently funded by the Global Fund must be fully integrated and operated by the national program and funded through the national budget.

To facilitate program transition, technical, managerial, and cost elements of programs need to be aligned with government norms.

Experiences from other countries show that when external support is withdrawn, surveillance data production and dissemination deteriorate. A striking example is Croatia, which hosts a WHO collaborative center for HIV/AIDS surveillance; however, after the Global Fund transition, HIV/AIDS reporting became irregular, leaving the international community with a knowledge gap on HIV within KPs.^56^ Thus during the transition, it becomes essential not only to shift funding onto the government, but also to ensure that critical information is still being generated and broadly disseminated.

It is essential to ensure that critical surveillance information is still being generated and broadly disseminated during and after transition.

The multiplicity of transition risks illustrated in this study highlights the need for further enhancement of national capacity through the provision of technical assistance and management support. The shift of health program responsibility from donor to program recipient means that the capacity previously supplied by donors must be replaced and/or adapted in line with the priorities and capacities of local actors.^32^^,^^52^ Therefore, as transition progresses it will require a steady reduction of investments in commodities to redirect the focus of donor resources to technical assistance and support, which should be adequately budgeted and funded during and after the transition.^10^ Based on our findings, technical support areas could range from supporting legislative amendments and enhancing advocacy efforts to improving management and stewardship capacity and procurement and supply management systems. The needs for technical assistance will vary from country to country, although some common areas will emerge, such as CSO/NGO contracting or amending national legislation/regulations to facilitate the uninterrupted supply of quality-assured drugs and commodities.

Finally, monitoring transition is essential; it must start before the transition begins and should follow the entire process. The approach to monitoring might differ depending on the purpose it has to serve. Examples include monitoring the course to correct the transition process and ensure that activities are adapted to emerging changes; obtain evidence necessary for advocacy with donors or hold governments accountable; and keep key stakeholders informed and engaged in the transition process. Studies^6^^,^^16^^,^^52^ suggest various approaches and tools for monitoring the transition process, but all agree that the system and tools cannot be one size fits all; they must be adjusted to the purpose and the context. The monitoring system must include quantitative indicators and qualitative investigation as complementary approaches because both are necessary to fully explore transition and actively engage community and/or civil society representatives in the transition monitoring process. Monitoring must have adequate resources to deliver on its objectives, and monitoring results must be broadly shared with the national and international stakeholders involved with the country.

Monitoring transition is essential; it must start before the transition begins and should follow the entire process.

### Limitations

Our study has several limitations. First, this paper is based in part on a limited number of prospective evaluations of donor transitions. Consequently, we encountered a lack of commonly agreed theoretical frameworks for evaluating the transition process. We tried to address this limitation by using a single theoretical framework to systematically collect comparable information across countries. Moreover, information obtained from publicly available documents was validated and/or complemented by stakeholder interviews to arrive at balanced and well-triangulated findings. Secondly, our TPA framework, while based on a thorough literature review, is partly informed by the collective practical experiences of the authors and has not been used prospectively in any program transition, beyond this study. Finally, we focused our research efforts on exploring the main program areas exposed to the transition risks. Exploring some issues in more depth would have afforded richer insights; however, due to the scale of the research and funding limitations, such exploration was not possible. Nevertheless, the similarities observed in our country samples and their associated risks resonate with findings from other programs, which gives us confidence that the findings from this study can be broadly shared.

## CONCLUSIONS

To manage transitions responsibly, the Global Fund needs to fully and strategically exploit its funding and partnership model at the global, regional, and local levels.

First, it is essential for the Global Fund Board to fully understand transition risks and allow for a gradual transition process over time that is linked not only to GDP per capita and disease burden but also to other important and measurable indicators. Such indicators obviously need to be elaborated and reflected during the sustainability transition and in cofinancing, and probably in the eligibility policy and potentially in the allocation formula as well.^57^^,^^58^ However, the appetite of the Global Fund Board to consider other elements beyond GDP and disease burden or to revise allocation formula is not reflected in its decisions or documents.

Second, the Global Fund has to develop its capability to negotiate a transition with the country government, and not only with the CCM, and to enforce the negotiated agreements. For the time being, Global Fund policies do not reveal a readiness to explore alternative mechanisms for legal engagement with sovereign governments instead of CCMs.

Third, the Global Fund's approach to transition does not seem to be sufficiently nuanced to ensure gradual and smooth transitions. Numerous concerns exist across the range of stakeholders, which indicates a need for closer attention and eventual reflection on the part of the Global Fund about the complexities related to transition in the Board's established policies and/or in guidance notes issued by the Global Fund secretariat.

Fourth, since the Global Fund is a partnership model without a country presence, it needs to strategically exploit the competencies and advantages of its partners to provide tailored technical assistance and support to countries. For some transition challenges, the support could be delivered at a regional level (for similar problems across the country group) by engaging a regional partner through a partnership arrangement. Examples include involving WHO to help countries improve national drug legislation in a way that facilitates the supply of quality drugs and commodities; enhancing procurement planning capabilities through regionally delivered training workshops and/or tools; and applying a regional grant-making mechanism involving capable regional watchdog organizations that use a regional platform for country-level operations. The organization would then be able to hold national governments accountable for their promises in the transition plan or involve advocates to push for specific changes in the policy and/or practice also spelled in the transition plan.

Finally, the Global Fund could strategically exploit the capabilities of partners that have a more significant presence or ability to deliver assistance at the country level, such as the German BACKUP initiative, France's 7%, PEPFAR, USAID, and others. In close cooperation with such a partner, the Global Fund could identify country-specific areas for support and seek their assistance in helping country governments implement the required changes.

To manage transitions responsibly, the Global Fund needs to exploit its funding and partnership model at the global, regional, and local levels.

To conclude, only concerted efforts on the part of the Global Fund and its partners, coupled with well-planned transition and coordinated support to the countries involved, can ensure a smooth and sustainable transition without undermining the public health gains achieved with donor help. However, this transition cannot be achieved unless the Global Fund Board and especially its donor constituency fully recognize the complexities of transition and demonstrate readiness to take the bold steps necessary to revise transition policy and procedures.

## Supplementary Material

18-00425-Sulaberidze-SupplementTable2.pdf

18-00425-Sulaberidze-SupplementTable1.pdf

## Notes

**Peer Reviewed**

**Funding:** The authors report financial support from the Global Fund and UNAIDS while conducting of the country case studies. Preparation of the article was financially supported by Curatio International Foundation.
