# Supplementary material for: The Challenges of Transition From Donor-Funded Programs: Results From a Theory-Driven Multi-Country Comparative Case Study of Programs in Eastern Europe and Central Asia Supported by the Global Fund
Source: Glob Health Sci Pract. 2019 Jun 24;7(2):258–72. doi: 10.9745/GHSP-D-18-00425 (PMC6641812; doi:10.9745/GHSP-D-18-00425)
Supplement: 18-00425-Sulaberidze-SupplementTable2.pdf [file 18-00425-Sulaberidze-SupplementTable2.pdf]

Gotsadze G, Chikovani I, Sulaberidze L, Gotsadze T, Goguadze K, Tavanxhi N. The challenges of transition from donor-funded programs: results from a theory-driven multi-country comparative case study of programs in Eastern Europe and Central Asia supported by the Global Fund. *Glob Health Sci Pract*. 2019;7(2).
https://doi.org/10.9745/GHSP-D-19-00425

SUPPLEMENT TABLE 2. Comparative Table of Risk to Transition Across the Countries[a]

			**Country and Year of Assessment**									
**TPA Component**	**TPA Indicator**	**Risk Category/ Disease**	**ARM 2017**	**BLR 2015**	**BGR 2015**	**GEO 2015**	**KOS 2017**	**KGZ 2016**	**MDA 2016**	**TKM 2017**	**UKR 2015**	**UZB 2017**
**External Environment**												
Economic environment	Favorable economic indicators (increasing GDP per capita and share of general government revenues as % of GDP)	HIV/TB	M	L	L	L	H	M	M	M	M	M
Political environment	Existence of political will to prioritize health investments	HIV/TB	M	L	L	M	H	M	L	L	M	L
	Existence of laws, regulations, or policies that hinder effective prevention, treatment, care, and support for KPs and people living with diseases; Rule of Law	HIV	H	H	M	M	H	M	H		M	H
		TB	M	H	M	M	M	M	M	M	M	M
	Legislation conducive for social contracting; CSO contracting practiced in any sector	HIV/TB	L	H	L	L	L	L	M	H	L	L
**Internal Environment**												
Inputs												
Financing		HIV	H	M	M	M	H	H	H		H	H

Gotsadze G, Chikovani I, Sulaberidze L, Gotsadze T, Goguadze K, Tavanxhi N. The challenges of transition from donor-funded programs: results from a theory-driven multi-country comparative case study of programs in Eastern Europe and Central Asia supported by the Global Fund. *Glob Health Sci Pract*. 2019;7(2).
https://doi.org/10.9745/GHSP-D-19-00425

			Country and Year of Assessment									
**TPA Component**	**TPA Indicator**	**Risk Category/ Disease**	**ARM 2017**	**BLR 2015**	**BGR 2015**	**GEO 2015**	**KOS 2017**	**KGZ 2016**	**MDA 2016**	**TKM 2017**	**UKR 2015**	**UZB 2017**
	Budgetary commitment to disease (increasing public expenditure and share on disease program)	TB	H	M	L	H	H	H	M	L	H	L
	Prevention priority (increasing public expenditure and share on prevention)	HIV	H	H	H	H	H	H	H		H	H
	Treatment financing from public sources (second-line drugs for TB)	HIV	H	M	M	M	H	H	L		M	M
		TB	H	H	H	H	H	H	H	M	H	H
	Prevention intervention (low threshold services, OST) financing from public sources	HIV	H	H	H	H	H	H	H		H	H
Human resources	Sufficient human resources (quantities, geographic distribution, and aging)	HIV	L	H	L	M	L	M	M		L	M
	Institutionalization of donor-supported training programs; existence of policy for production/training of CSO personnel; donor-funded HR salaries aligned with national pay scale	TB	M	M	M	M	M	M	M	M	M	M
		HIV	H	H	H	H	H	M	M		H	M
		TB	H	H	H	H	H	M	H	M	H	M
Health information systems	Routine statistical reporting—integration in the national system	HIV	L	L	M	L	M	M	L		L	M
		TB	L	M	M	L	M	M	L	M	M	L
	Routine statistical reporting—level of advancement	HIV	M	M	M	M	M	M	L		M	H
		TB	M	M	M	L	M	M	L	H	M	M

Gotsadze G, Chikovani I, Sulaberidze L, Gotsadze T, Goguadze K, Tavanxhi N. The challenges of transition from donor-funded programs: results from a theory-driven multi-country comparative case study of programs in Eastern Europe and Central Asia supported by the Global Fund. *Glob Health Sci Pract*. 2019;7(2). https://doi.org/10.9745/GHSP-D-19-00425

			Country and Year of Assessment									
**TPA Component**	**TPA Indicator**	**Risk Category/ Disease**	**ARM 2017**	**BLR 2015**	**BGR 2015**	**GEO 2015**	**KOS 2017**	**KGZ 2016**	**MDA 2016**	**TKM 2017**	**UKR 2015**	**UZB 2017**
	HIV second-generation surveillance—methodologies, timeliness	HIV	L	L	H	L	L	L	L		L	L
	HIV second-generation surveillance—funding from public sources	HIV	H	H	H	H	H	H	H		H	H
Governance												
Governance	Political commitment to diseases	HIV	M	M	M	H	H	M	L		L	H
		TB	M	M	L	M	M	M	L	L	H	H
	Strong coordination mechanisms	HIV/TB	M	M	M	M	H	M	M	M	H	M
Program												
CSO contracting practice	CSOs contracting in health	HIV/TB	M	H	H	M	M	H	M	H	H	H
Organizational capacity	Procurement and supply management	HIV/TB	H	M	L	L	M	M	L	H	H	M

Abbreviations: ARM, Armenia; BLR, Belarus; BGR, Bulgaria; CSO, civil society organization; GDP, gross domestic product; GEO, Georgia; HR, human resources; KOS, Kosovo; KGZ, Kyrgyzstan; MDA, Moldova; OST, opioid substitution therapy; TB, tuberculosis; TKM, Turkmenistan; TPA, transition preparedness assessment; UKR, Ukraine; UZB, Uzbekistan.

[a] Red color *or* H, high risk for transition (many or significant barriers exist); yellow color *or* M, moderate risk for transition (few barriers exist); green color *or* L, low risk for transition (absence of major barriers). Cells that are blank (no color or letter) indicate that the topic was not studied.
