# Supplementary material for: The Challenges of Transition From Donor-Funded Programs: Results From a Theory-Driven Multi-Country Comparative Case Study of Programs in Eastern Europe and Central Asia Supported by the Global Fund
Source: Glob Health Sci Pract. 2019 Jun 24;7(2):258–72. doi: 10.9745/GHSP-D-18-00425 (PMC6641812; doi:10.9745/GHSP-D-18-00425)
Supplement: 18-00425-Sulaberidze-SupplementTable1.pdf [file 18-00425-Sulaberidze-SupplementTable1.pdf]

Gotsadze G, Chikovani I, Sulaberidze L, Gotsadze T, Goguadze K, Tavanxhi N. The challenges of transition from donor-funded programs: results from a theory-driven multi-country comparative case study of programs in Eastern Europe and Central Asia supported by the Global Fund. *Glob Health Sci Pract*. 2019;7(2). https://doi.org/10.9745/GHSP-D-19-00425

SUPPLEMENT TABLE 1. Number of Country Representatives Participating in Transition Preparedness Assessment

Countries	State Institutions and National HIV and TB Program Representatives	Donors and Partners	Local CSO, Professional Associations
Armenia	12	5	4
Belarus	18	3	3
Bulgaria	21	2	11
Georgia	17	6	14
Kosovo	13	2	6
Kyrgyzstan	12	5	2
Moldova	16	6	5
Turkmenistan	6	5	12
Ukraine	15	12	7
Uzbekistan	14	4	6

Abbreviations: CSO, civil society organization; TB, tuberculosis.
